# Reproductive traits affect the rescue of valuable and endangered multipurpose tropical trees

**DOI:** 10.1093/aobpla/plw051

**Published:** 2016-08-02

**Authors:** Viviane Sinébou, Muriel Quinet, Bonaventure C. Ahohuendo, Anne-Laure Jacquemart

**Affiliations:** ^1^Research Group Genetics, Reproduction, Populations, Earth and Life Institute – Agronomy, Université Catholique de Louvain, Croix du Sud 2 Box L7.05.14, B-1348 Louvain-la-Neuve, Belgium; ^2^Département de Productions Végétales, Faculté des Sciences Agronomiques, Université Abomey-Calavi, Cotonou 01 BP 526, Benin

**Keywords:** Bees, breeding system, conservation biology, mating system, pollinator efficiency, seed germination

## Abstract

Conservation strategies are urgently needed in Tropical areas for widely used tree species. Increasing numbers of species are threatened by overexploitation and their recovery might be poor due to low reproductive success and poor regeneration rates. One of the first steps in developing any conservation policy should be an assessment of the reproductive biology of species that are threatened by overexploitation. This work aimed to study the flowering biology, pollination and breeding system of *V. doniana*, a multipurpose threatened African tree, as one step in assessing the development of successful conservation strategies. To this end, we studied (1) traits directly involved in pollinator attraction like flowering phenology, flower numbers and morphology, and floral rewards; (2) abundance, diversity and efficiency of flower visitors; (3) breeding system, through controlled hand-pollination experiments involving exclusion of pollinators and pollen from different sources; and (4) optimal conditions for seed germination. The flowering phenology was asynchronous among inflorescences, trees and sites. The flowers produced a large quantity of pollen and nectar with high sugar content. Flowers attracted diverse and abundant visitors, counting both insects and birds, and efficient pollinators included several Hymenoptera species. We detected no spontaneous self-pollination, indicating a total dependence on pollen vectors. *Vitex doniana* is self-compatible and no inbreeding depression occurred in the first developmental stages. After extraction of the seed from the fruit, seed germination did not require any particular conditions or pre-treatments and the seeds showed high germination rates. These pollination and breeding characteristics as well as germination potential offer the required conditions to develop successful conservation strategies. Protection, cultivation and integration in agroforestry systems are required to improve the regeneration of the tree.

## Abstract (precis)

Conservation strategies are urgently needed for widely used tree species. Increasing numbers of species are threatened by overexploitation and their recovery might be poor due to low reproductive success, and poor regeneration rates. One of the first steps for any conservation policy should be an assessment of their reproductive biology. We studied the flowering biology, pollination, breeding system and germination potential of *Vitex doniana*, a multipurpose threatened African tree. *Vitex doniana*'s breeding characteristics as well as germination performance offered the required conditions to develop successful conservation strategies. Protection and integration in agroforestry systems could improve the regeneration of the tree.

## Introduction

Global biodiversity is decreasing at an unprecedented rate as a complex response to several human-induced changes (Sala *et al.* 2000). Land use change is the driver that has the largest global impact on biodiversity, mostly due to habitat destruction and fragmentation. Land use change is particularly the most important driver in tropical forests (Sala *et al.* 2000). The African forests are subject of haphazard modification following anthropogenic pressures including tree cutting and clearing for agriculture. In West Africa, logging and seasonal fires set by farmers and hunters have increased deforestation, destroying an average of 870 000 ha/year between 2000 and 2010 (FAO 2011). Consequently, several food and medicinal tree species have been declared locally endangered and are priorities for conservation (Eyog-Matig *et al.* 2002; FAO 2011). Forest harvesting directly decreases the rates of survival, growth and reproduction of individual trees, affecting the structure and dynamics of harvested populations (Oumorou *et al.* 2010). In harvested stands, only large, old trees and a few seedlings and saplings generally survive (Oumorou *et al.* 2010). Maintaining these local remnant populations will require informed management and conservation practices.

*Vitex doniana*, commonly called black plum, is one of the most important wild-harvested, multipurpose trees in tropical Africa (Akoègninou *et al.* 2006; N’Danikou *et al.* 2015). The tree is widely used for food, medicinal purposes and as firewood (Achigan-Dako *et al.* 2011). The species also has a great socio-cultural and mythological importance for local people (Dadjo *et al.* 2012; N’Danikou *et al.* 2015).

Despite its high value for local populations, there is no evidence of any conservation initiatives from harvesters (N’Danikou *et al.* 2011). People usually harvest the desired resources without considering regeneration and management or attempting silvicultural practices (e.g. planting and sowing) (Dadjo *et al.* 2012; N’Danikou *et al.* 2015). Mainly due to overexploitation, *V. doniana* is one of the most-threatened food tree species with high priority for conservation in Benin, Kenya, Niger and Burkina Faso (Eyog-Matig *et al.* 2002; Oumorou *et al.* 2010; N’Danikou *et al.* 2015).

The threats to the tree affect multiple reproductive processes. Even, if the species is partially spared during land clearing, because of its many uses and high market value (Dadjo *et al.* 2012; N’Danikou *et al.* 2015), fruit harvesting reduces available seeds for recruitment, and overexploitation of the leaves, branches, bark and wood increases the pressures on remnant populations (Oumorou *et al.* 2010). Also, seed germination creates a bottleneck due to dormancy, which hampers the species’ natural regeneration (N’Danikou *et al.* 2011). *Vitex doniana* has a hard seed coat, reducing germination rates, and seedlings are scarce in natural habitats due to fires and grazing pressure. Moreover, leaf harvest reduces reproductive success by delaying flowering and decreasing seed set (Dadjo *et al.* 2012; N’Danikou *et al.* 2015). Therefore, a sustainable conservation strategy is urgently needed to preserve this valuable tree.

Successful conservation depends on sexual reproduction and seedling recruitment (Vodouhè *et al.* 2011). The failure of reproductive processes such as pollination often causes species loss (Faegri and van der Pijl 1979; Moza and Bhatnagar 2007). The failure of pollination has diverse causes, including pollinator rarity or infidelity, poor quantity or quality of pollen deposited on the stigmas, delayed stigma receptivity and self-incompatibility (Faegri and van der Pijl 1979). Other key factors of plant reproductive biology include floral phenology, the periodicity in the production of flowers, which follows a species-specific schedule, ranging from complete synchrony within the population to full asynchrony, thus shaping mating possibilities among individuals (Ewédjè *et al.* 2015). The breeding system also shapes exchanges among individuals, mainly by self-incompatibility (Faegri and van der Pijl 1979; Kalinganire *et al.* 2000).

Despite its importance for conservation, the reproductive biology of *V. doniana* remains unexplored. Thus this work aimed to study the flower biology, pollination, and breeding system of *V. doniana* and to assess reproductive outcomes to develop successful conservation strategies. To this end, we studied traits that directly influence reproductive success including (1) flowering phenology, flower numbers and morphology, and floral rewards; (2) abundance, diversity and efficiency of flower visitors; (3) breeding system; and (4) seed germination.

## Methods

### Study species and studied sites

**Figure 1.**
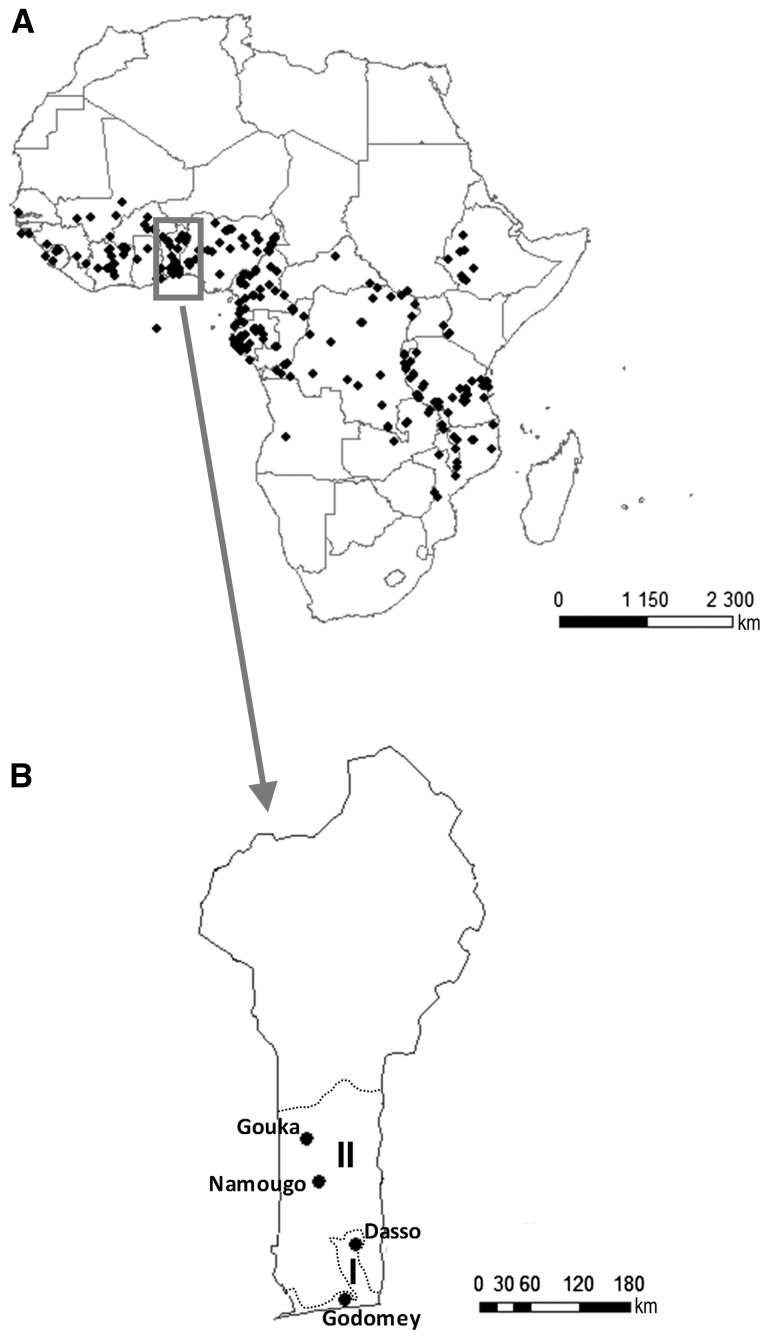
Spatial distribution of *Vitex doniana*. (A) Africa: map based on geographical coordinates from herbarium specimens from the Botanical Gardens of Meise, Université Libre de Bruxelles, Wageningen and Leiden (October 2015); (B) The four studied populations in Benin. Agroecological zones are adapted from Ky (2008): (I) Guineo-Congolian zone and (II) Guineo-Sudanian transition zone.

The genus *Vitex* (Lamiaceae) includes over 270 species, predominantly trees and shrubs, and is mainly present in tropical and subtropical regions (Eyog-Matig *et al.* 2002). *Vitex doniana* is the most widespread species in the genus (Ky 2008). This medium-sized (8–20 m tall) deciduous tree grows in coastal woodlands, riverine and lowland forests, and savannahs of sub-Saharan Africa, east from Senegal to Somalia and south to South Africa (Kapooria and Aime 2005; Ky 2008; Dansi *et al.* 2008; Maundu *et al.* 2009; Dadjo *et al.* 2012; N’Danikou *et al.* 2015; Fig. 1). The tree is widely used for food, medicinal purposes and as firewood (Achigan-Dako *et al.* 2011). Processing the fleshy fruits provides juice, jam, syrup and alcohol (Agbede and Ibitoye 2007; Mapongmetsem *et al.* 2005, 2008; Oumorou *et al.* 2010; Ajenifujah-Solebo and Aina 2011; Vunchi *et al.* 2011; Dadjo *et al.* 2012). Also, new shoots and leaves are traditionally consumed as leafy vegetables (Codjia *et al.* 2003; Dansi *et al.* 2008; Achigan-Dako *et al.* 2011), and almost all parts of the plant have medicinal properties (anti-bacterial, anti-inflammatory, analgesic, anti-fungal, hepato-protective, anti-oxidant and hypotensive; Ladedji and Okoye 1996; Ladedji *et al.* 1996; Ganapaty and Vidyadhar 2005; Iwueke *et al.* 2006; Kilani 2006; Padmalatha *et al.* 2009; Lagnika *et al.* 2012; Adetoro *et al.* 2013; Hamzah *et al.* 2013). Overall, 25 diseases are known to be treated with Black plum (Oumorou *et al.* 2010; Dadjo *et al.* 2012). The wood is used for firewood, charcoal, and furniture.

Our observations and experiments were conducted on four sites situated in Southern and Central Benin, West Africa (Fig. 1 and Table 1). The Godomey and Dasso sites, in the South of Benin, were located in the Guineo-Congolian climatic zone, which is characterized by two rainy seasons. The Guineo-Congolian zone is located between latitudes 6°25′N and 7°30′N. Total mean annual rainfall averaged 1200 mm. These sites have relative humidity of 69–97 % and mean daily temperatures of 25–29 °C. The Namougo and Gouka sites were in the Guineo-Sudanian climate regime which is characterized by one rainy season (from mid-April to October), approximately 7 months of dry season, annual rainfall of 900–1200 mm and mean daily temperatures of 19–36 °C (Yédomonhan *et al.* 2012).

**Table 1.** *Vitex doniana* studied site location: geographical coordinates, agroecological zones, main crops on the sites, type of sites, leaf collection intensity and distance among trees per site.

Sites	Geographical coordinates	Agroecological zones	Main crops	Type of site	Exploitation intensity	Distance among trees (m)
Godomey	6°25’15.9’’ N2°20’48.4’’E	Guineo-Congolian	Palm tree, maize, cassava, cowpea	Farm	Young leaves poorly collected	50–2000
Dasso	7°00’42.8’’N2°27’59.5’’E	Guineo-Congolian	Palm tree, maize, cassava, peanut	Farm	Branches hardly cut, young leaves regularly collected (∼70 %)	10–200
Namougo	7°41’38.2’’N2°04’32.8’’E	Guineo-Sudanian	Maize, cassava, yam, peanut	Woodland	Branches cut but foliage in recovery from overexploitation one year before	10–1500
Gouka	8°09’23.7’’N1°56’09.3’’E	Guineo-Sudanian	Maize, cassava, yam, peanut	Degraded forest gallery	Branches cut but foliage in recovery from overexploitation one year before	10–200

All trees were located in agricultural landscapes, in close proximity to farms and traditional fields of maize, cassava, and peanut, with palm trees (in the Guineo-Congolian zone) or yams (in the Guineo-Sudanian zone).

The trees in Namougo and Gouka sites were protected, as leaf collection has been forbidden since 2013. Leaf collection occurred before and during the study at the Godomey and Dasso sites.

### Phenology and floral synchrony

**Figure 2.**
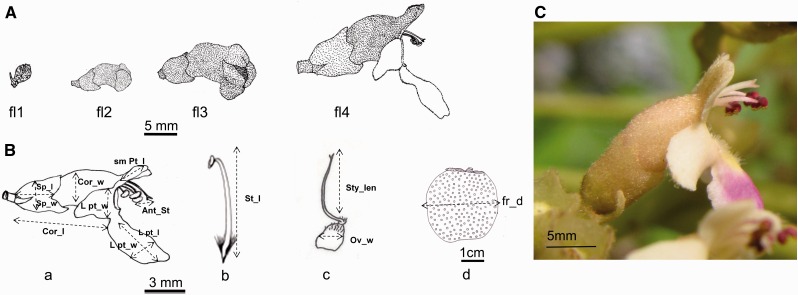
Flower morphology of *Vitex doniana.* (A) Phenological stages: fl1 – young bud, fl2 – well-developed bud, fl3 – flower at anthesis, fl4 – flower 1 day after anthesis. (B) Measured floral and fruit traits: (a) flower traits, (b) androceum, (c) gynoecium, (d) fruit. Cor_l: corolla length, Sp_w: Sepal width, Sp_l: Sepal length, sm pt_l: small fused petal length, L pt_w: lateral fused petal width, L pt_w: lower petal width, L pt_l: lower petal length, Ant_St: anther stigma distance, St_l: stamen length, Sty_len: style length, Ov_w: ovary width, Fr_d: fruit diameter. (C) Photograph of the flower showing the relative position of the stigma and the anthers.

We randomly sampled 10 adult trees per site during the flowering and fruiting periods over two successive years (2014 and 2015). Flowering was observed weekly, from January to May, and fruit development was observed every 2 weeks on the same individuals, from February to April. Four stages were defined for flowering: young bud (fl1), well-developed bud (fl2), flower at anthesis (fl3) and flower withering (fl4, Fig. 2A). Two stages were defined for fruiting: fruit initiation (persistent expanded ovary, fr1) and young green fruits (fr2).

Floral phenology was followed daily on 20 flowers between stage fl2 and fl4 per tree on each site for 2 weeks to assess the flower life span and dichogamy (protogyny or protandry).

The quantity of flowers and fruits in a given stage per tree was quantified visually by using binoculars to inspect all branches. Data were collected in ordinal classes: 0 = absence of flowers or fruits in a given stage; 1= 1–25 % of branches bearing organs in a given stage; 2= 26–50 %; 3= 51–75 %; 4= 76–100 %. The dates of first flowering and the duration of each stage were recorded to construct the temporal development of phenophases at each site.

### Flower number and morphology

Four inflorescences per branch (on 4–11 branches per tree depending on the tree size) from five trees per site were examined to determine the mean number of flowers per inflorescence. The number of flowers per tree was extrapolated for each tree by multiplying numbers of flowers per inflorescence by the numbers of inflorescences per branch and the numbers of branches per tree.

Eighty inflorescences, four inflorescences per tree and per site, were dissected to determine whether all flowers were hermaphroditic, to describe the structure of the androecium and the gynoecium, and to document the number of stamens and ovules and the number of nectaries per flower. The flower colour was also observed.

Two hundred flowers (randomly selected from two different inflorescences from 20 trees, five trees per site) were dissected to describe their structure. A caliper was used to measure the length and width of the sepals, corolla tube, lateral fused petals, inferior petal and small fused superior petals. We also measured the lengths of the exterior and inner cycles of stamens, the diameter and length of the ovary, and the distances between the stamens and the stigma (Fig. 2B).

For quantification of pollen grains, 20 anthers were collected from ten flowers buds (fl2) per tree and per site. Pollen grains were counted under a light microscope (AO Spencer). Anthers were individually crushed in a microcentrifuge tube containing 100 µL of Alexander’s stain (Kearns and Inouye 1993), mixed and sonicated to homogenize the pollen grains into the solution. For each anther, the number of pollen grains in 10 µl was counted in triplicate under a light microscope (AO Spencer). To assess pollen viability, one anther per flower stage was collected from each of 10 flower buds (fl2) or flowers (fl3) the morning and afternoon of the day of anthesis and (fl4) the day after anthesis from 10 different trees in each site (200 anthers in total). Pollen grains from one anther were then dispersed by squeezing on a glass slide in a drop of Alexander’s solution. A minimum of 200 pollen grains were observed per anther in triplicate and counted according to their colour. Viable grains had a red protoplasm, whereas aborted grains were empty exhibiting a green colouration in their wall. Percentage of pollen viability was calculated based on the number of viable and total grains.

Stigma receptivity was estimated by fruit initiation (persistent expanded ovary) following hand pollinations on a total of 120 flower buds or flowers from three developmental stages (fl2, fl3 and fl4) on the five study trees in each site (*N* = 2 per stage and per tree).

For the estimation of nectar production, 100 flowers from five different trees per site were covered with exclusion bags (Delnet pollination bags, USA) 24 h before sampling to prevent any visit. Nectar production was assessed twice a day (morning and afternoon) on the same flowers. Nectar was collected with 5 μL glass capillary tubes (Hirschmann Laborgerate, Eberstadt, Germany), and nectar volume was estimated by measuring the length of the nectar column in the capillary tube. Sugar concentration was measured with a low-volume hand refractometer (Eclipse Handheld refractometer, Bellingham and Stanley Ltd, Tunbridge Wells, UK) and expressed as the percentage of sucrose (w/w). To determine the total sugar content of nectar per flower, sugar concentration (%) was converted to mg/µL according to the following formula: *y* = 0.00226 + (0.00937*x*) + (0.0000585*x*^2^) where *y* is the sugar concentration (mg/µL) and *x* is the sugar concentration (%) (Dafni *et al.* 2005). The total sugar content of nectar per flower (mg) was then calculated as volume of nectar (µL) × sugar concentration (mg/µL).

The sugar composition of the nectar was determined by high-performance liquid chromatography with a Shimadzu HPLC system coupled to a RID10A refractometer (Shimadzu, ‘s-Hertogenbosch, Netherlands) using a Hypersil gold amino (150 × 4.6 mm) column (Thermo Scientific, Aalst, Belgium) at 26 °C. The mobile phase consisted of 83 % acetonitrile in water and the flow was 1.0 mL min^−1^. Analyses for nectar composition were performed in the Groupe de Recherche en Physiologie Végétale (Université catholique de Louvain, Louvain-la-Neuve, Belgium).

### Floral visitors and their pollination potential

On two trees per site, four inflorescences per tree were marked for observations of insect and bird visitors. Insects and birds visiting flowers were observed from January to May 2014 for five consecutive days per site. The observations were conducted at 0700, 0900, 1100, 1400 and 1700 h, for 5 min per inflorescence for each time period. A total of 17 h of observations was performed at each site.

Visitor abundance and behaviour (nectar and/or pollen collection, and contacts with reproductive organs) were assessed. Insects that were not identified in the field were collected by hand net, killed with ethyl acetate, identified and stored separately in small vials. Precise identifications were performed at the Centre régional de Biodiversité des insectes from the Institut International d’Agriculture Tropicale (Abomey-Calavi, Benin) and at the Institut Royal des Sciences Naturelles de Belgique (Brussels, Belgium).

The potential of the different insect visitors to be effective pollinators was estimated by determining their relative abundance, fidelity and capacity to carry pollen. Their fidelity was indirectly estimated by the proportion of pollen from *V. doniana versus* other plant species in their corbicular pollen loads. Their capacity for carrying pollen was directly assessed by counting the pollen grains on the different parts of the insect body. For insect fidelity, in each site, insects captured with pollen loads were immobilized in a bee-marking cage and one pollen load was removed by toothpick per individual. For the four sites, a total of 60 pollen loads was collected and acetolyzed (Erdtman 1960, modified). From each sample, approximately 400 randomly chosen pollen grains were identified by light microscopy (Leitz Wetzlar). Pollen identification was based on a reference slide of *V. doniana* pollen. A total of 13 insect species per site (20 individuals per species) were examined for their carrying capacity. For each visitor type, pollen identity and quantity on insect bodies were assessed. The pollen grains were removed from the different insect body parts (head, abdomen and legs) using a small cube of gelatin passed over each part of the insect body. The gelatin was melted and observed under a light microscope (Ernst Leitz Wetzlar).

### Breeding system experiments

To assess the breeding system, 1600 flowers were marked and allocated to one of the four pollination treatments. Each treatment was performed on five flowers per inflorescence, four inflorescences per tree and five trees in each of the four sites. All hand-pollinated flowers were emasculated at bud stage.

One treatment was carried out in the presence of pollinators (T1; free exposure for control or open pollination). Two treatments involved hand-pollinations: (T2) self-pollination with pollen from another flower of the same tree, to test for self-compatibility and (T3), cross-pollination with pollen from another tree in the same site, to assess inbreeding depression when comparing with T2. To quantify spontaneous self-pollination, flowers in the fourth treatment (T4) were bagged and left unmanipulated.

Except for the control treatments, all flowers were bagged before flower anthesis to exclude visitors (Delnet pollination bags, USA). All bags were removed at the start of fruit maturation. Pollination was carried out by brushing anthers of the donor flower on the stigma of the recipient flower. Hand pollinations were performed daily from 0700 to 1100 h.

### Pollen germination and pollen tube growth

Following hand-pollination treatments, 10 pistils were removed at different times after pollination (15 and 30 min, 1, 2, 4, 6, 24, and 48 h). Pistils were fixed in FAA (ethanol 70 %: formaldehyde 35 %: acetic acid; 8:1:1) and stored at room temperature. Before observation, the pistils were rinsed with distilled water, softened and clarified in 4M NaOH for 4 h at room temperature. The pistils were rinsed again and stained for 2 h in 0.1 % aniline blue solution in 0.1M KH_2_PO_4_ (Kearns and Inouye 1993). Pollen germination on stigmas and pollen tube growth in the styles were examined under a fluorescence microscope (Nikon Optiphot-2/LH-M100C-1) with a 420-nm to 440-nm excitation filter and a 480-nm emission filter, according to Kearns and Inouye (1993). The numbers of pollen grains per stigma, of pollen grains initiating a pollen tube and of pollen tubes reaching the end of the style were counted.

### Fructification and germination

Mature fruits were harvested 4 months after pollination. Fruit set (percentage of flowers giving mature fruits), fruit fresh weight, numbers of viable seeds, weight of 100 viable seeds and germination rate were measured.

In September 2013, a preliminary *ex situ* germination test was performed under controlled conditions on 240 ripe fresh fruits. Fruit samples were divided into batches of 10 fruits for germination treatments. Besides control batches (T0), three pre-treatments were tested: soaking in distilled water for 24 h (T1), soaking in sulfuric acid 95 % for 1 h and rinsed with demineralized water (T2), and scarification with a scalpel (T3). Six replicates of 10 fruits per treatment were put on filter paper in jars (300 mL volume), watered with demineralized water and over-wrapped with a plastic film. They were then placed in two germination chambers (Snijders Scientific, Netherlands). Two temperature regimes were then applied to the seeds (30/25 °C or 35/30 °C with a photoperiod cycle 12 h dark: 12 h light). Temperature conditions were chosen since they correspond to the soil temperature in the natural environment of *V. doniana* (N’Danikou *et al.* 2014). Germination was recorded once a week during 6 months. No germination was observed and the test was stopped due to fungal infection.

In September 2014, germination tests were performed *in situ* following hand pollination treatments on 10 samples of five seeds for hand self- and cross-pollinated fruits. Extracted dried seeds were sown in 30 black LDPE (low-density polyethylene) nursery bags (29.6 cm³) filled with ground soil. Bags were left under ambient conditions, and watered every 2 d. The total germination rate was assessed 45 d after sowing.

In September 2015, seeds were extracted from fruits and sun-dried for 10 h before being stored in paper bags until use. Seed samples were divided into four batches of 100 seeds for germination treatments under controlled conditions. Seeds were or were not submitted to a pre-treatment: soaking (S) or not (NS) in demineralized water for 2 h (Ahoton *et al.* 2011; N'Danikou *et al.* 2014). Two temperature regimes were then applied to the seeds (30/25 °C or 35/30 °C with a photoperiod cycle 12 h dark: 12 h light). Five replicates of 20 seeds per treatment were put on filter paper in petri dishes (90 mm diameter) and watered with demineralized water. They were then placed in germination chambers (Snijders Scientific, Netherlands). Seeds were considered germinated when the radicle protruded. Germination was recorded every 2 days. Final germination rate was estimated after 21 days, after several days without any new seedling emergence.

### Data analysis

Results were compared by analyses of variance (ANOVA, one- and two-way). Normality of the data was estimated with Shapiro–Wilk tests and homoscedasticity was verified using the Levene test. Data were transformed when required to ensure normal distribution and Welch’s correction was applied when homoscedasticity was not met. Differences among sites or conditions were assessed using Tukey’s HSD post-hoc tests.

Pearson correlations were performed among flower and fruit measurements. Significant correlations were detected among several floral traits: length and width of lateral fused petals (*R* = 0.24, *P* = 0.01), length and width of small fused superior petals (*R* = 0.81, *P* < 0.0001), lengths of the exterior and the inner cycles of stamens (*R* = 0.69, *P* < 0.0001), and diameter and length of ovary (*R* = 0.27, *P* = 0.0001). Thus, 10 independent measures were analyzed: width and length of sepals, width of the lateral fused petals, length and width of the inferior petal, corolla tube length, length of the long stamens, diameter of the ovary, length of the style, and the distance between the long stamens and the stigma. In the same way, fruit diameter and length were significant correlated (*R* = 073, *P* < 0.0001), and only diameter was presented.

Chi-square tests were used to analyze the proportion of visiting insects among sites. Differences in the composition of pollen loads and pollen carried by different visitor species were visually assessed with heatmaps (‘heatmap.2’ command, ‘gplots’ package).

The self-compatibility index (*SCI*) and self-fertility index (*SFI*) were calculated according to Lloyd and Schoen (1992). *SCI* determines the capacity of a plant to produce zygotes following self-pollination relative to that following outcrossing. It is calculated as the ratio between the fruit set (or seed set) produced after hand self-pollination and the fruit set (or seed set) produced after hand cross-pollination (Lloyd and Schoen 1992). *SFI* gives an estimation of the capacity of a plant to produce fruits and seeds without any pollen vector. It is calculated as the fruit set (or seed set) of autonomous self-pollination relative to that of hand cross-pollination (Kouonon *et al.* 2009). *SCI* values above 0.2 indicate self-compatibility and *SFI* values above 0.2 indicate autonomous selfing (Lloyd and Schoen 1992).

Levels of inbreeding depression at different developmental stages (fruit set, seed set and seed germination) were determined as the ratio between relative performance of selfed progeny (*ws*) and outcrossed progeny (*wc*) (*δ* = 1 − (*ws/wc*); Charlesworth and Charlesworth 1987). Values around 0 indicate no inbreeding depression.

All analyses were performed under R version 2.2.1 (R Development Core Team 2013). Data are presented as means ± standard deviation.

## Results

### Phenology and floral biology

**Figure 3.**
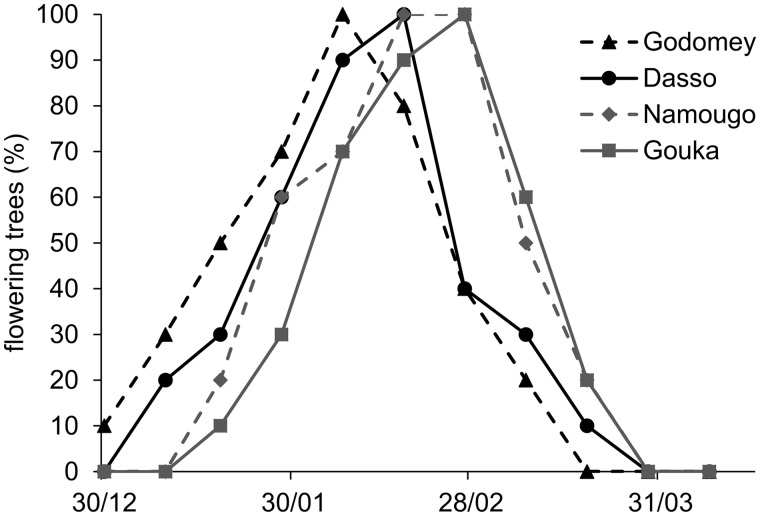
Percentage of *Vitex doniana* flowering trees in the four studied sites.

*Floral synchrony: Vitex doniana* flowered during the dry season, from December to April, and flowering was not synchronous among sites. Flowering began earlier in the Guineo-Congolian than in the Guineo-Sudanian sites: Godomey trees started and ended flowering first, while Namougo and Gouka trees initiated flowering 20–30 days later (Fig. 3A). Flowering was not synchronous within each site: flowering differences among trees were approximately 25 days, with differences especially pronounced in Dasso (41 days). Moreover, flowering was asynchronous within a tree, among inflorescences.

Flowers first appeared on new twigs, after leaves developed. After initiation, buds (fl1) reached stage fl2 (perianth developed, petals fused, buds elongated to 4-6 mm long x 2.6-2.8 mm wide) in 13–20 days. Stage fl3 with well-developed flower buds (central inferior petal deviated from the others, flower size of 7–9 mm long and 3–4 mm wide), occurred 22–28 days after bud initiation. At the fl3 stage, the flower opened as soon as any petal was touched, especially by insects or birds. The stage fl4 (all petals expanded, flowers 12–14 mm long and 3–4 mm wide) occurred 26–35 days after fl1. Flowers started to open in the morning between 0800 and 1200 h, with a high frequency around 1000 h. During this first step of anthesis, the anthers presented numerous white pollen grains along the longitudinal dehiscence splits. Stigmas reached receptivity with flower opening and remained receptive for 72 h. Flower life span (from opening to corolla drop) extended for 3–4 days.

Within an inflorescence, flowers displayed asynchronous anthesis: the terminal flower was the first that bloomed and it took 4–6 days for all flowers to open.

*Fruit maturation:* Fruit maturation was observed from the end of January to May, while fruits began to fall from June to September and fruit harvesting extended from July to September. The first signs of fruit development (fr1) were registered 5–7 days after corolla drop. At stage fr2 (21–34 days after fr1), young green fruits measured 10–15 mm wide. Fruits remained green until full maturity. The ripe fruits started to fall 102–141 days after fr2 (March to July). The colour only became black 5–8 days after lying on the ground. Fruit size and weight were significantly higher in Godomey than in other sites (Table 2).

**Table 2.** Morphological characteristics of inflorescences, flowers, and fruits of *vitex doniana* in the four studied sites (*n* = 200, means ±  SD).

Parameter	Godomey	Dasso	Namougo	Gouka	ANOVA/Kruskal- Wallis
Floral characteristics					
Flowers per tree	52938 ± 9786^a^	16322 ± 6738^b^	35635 ± 8396^ab^	43019 ± 4174^ab^	F = 5.23, *P* = 0.0104
Sepal length (mm)	4.01 ± 0.32^b^	3.98 ± 0.40^b^	4.19 ± 0.38^a^	4.10 ± 0.33^ab^	F = 3.31, *P* = 0.02
Sepal width (mm)	2.86 ± 0.34^a^	2.64 ± 0.24^b^	2.67 ± 0.25^b^	2.58 ± 0.15^b^	F = 11.39, *P* < 0.0001
Lateral fused petal width (mm)	3.35 ± 0.23^a^	3.65 ± 0.51^a^	4.10 ± 0.51^a^	3.58 ± 0.50^a^	F = 1.7, *P* = 0.19
Lower lip length (mm)	4.21 ± 0.48^a^	4.15 ± 0.47^a^	4.88 ± 0.53^a^	4.52 ± 0.84^a^	F = 1.8, *P* = 0.18
Lower lip width (mm)	7.52 ± 0.71^a^	7.14 ± 0.20^a^	7.02 ± 0.26^a^	6.90 ± 0.48^a^	F = 1.63, *P* = 0.2
Corolla tube length (mm)	7.34 ± 0.40^a^	7.29 ± 0.55^a^	6.73 ± 0.90^b^	6.65 ± 0.75^b^	F = 14.36, *P* < 0.0001
Ovary diameter (mm)	1.64 ± 0.20^a^	1.56 ± 0.24^b^	1.47 ± 0.17^c^	1.53 ± 0.16^bc^	F = 6.14, *P* = 0.0005
Stamen length (mm)	6.59 ± 0.50^a^	6.66 ± 0.54^a^	5.86 ± 0.54^c^	6.25 ± 0.66^b^	F = 20.69, *P* < 0.0001
Style length (mm)	7.49 ± 0.59^ab^	7.72 ± 0.70^a^	7.41 ± 0.64^b^	7.66 ± 0.41^a^	F = 2.97, *P* = 0.03
Anther-stigma distance (mm)	0.65 ± 0.15^a^	0.69 ± 0.12^a^	0.67 ± 0.17^a^	0.66 ± 0.11^a^	F = 0.84, *P* = 0.47
Pollen grains/anther	3903 ± 385^a^	3502 ± 391^a^	3952 ± 389^a^	3978 ± 364^a^	F = 0.64, *P* = 0.42
Fruit characteristics					
Fruit width (mm)	3.03 ± 0.37^a^	2.09 ± 0.25^b^	1.87 ± 0.24^c^	1.85 ± 0.23^c^	F = 382.94, *P* < 0.0001
Fruit weight (g)	16.33 ± 4.49^a^	6.01 ± 1.73^b^	4.92 ± 1.57^c^	4.78 ± 1.40^c^	F = 448.92, *P* < 0.0001

Means followed by different letters within a line are significantly different at *P* = 0.05.

*Inflorescence and floral biology:* Over all sites, inflorescences (dense and axillary cymes) averaged 79 ± 28 flowers. Based on the flower number per inflorescence and per tree, we calculated that a single tree produced between 16 300 (Dasso) and 52 900 (Godomey) flowers (Table 2). A significant proportion of flowers were white (30 %) at Godomey, but flowers were invariably purple at the other sites. Excepting anther-stigma distance and petal sizes, flower sizes differed significantly among sites (Table 2). Flowers from the Guineo-Sudanian sites (Namougo and Gouka) were significantly smaller than those from the Guineo-Congolian zone (Table 2). The number of pollen grains per flower did not differ among sites and averaged approximately 15 500 (Table 2). All flowers contained four ovules. At the flower morphology level, the two lobes of the stigma were divergent and faced the upper fused petals, whereas the dehiscent side of the anthers faced the lower petal, which could preclude spontaneous selfing (Fig. 2C).

**Figure 4.**
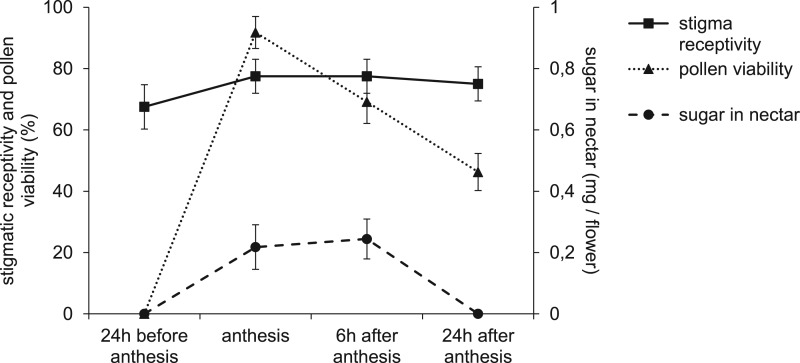
Stigma receptivity, pollen viability and sugar amount in the nectar of *Vitex doniana* ﬂowers for all sites pooled according to ﬂower age. Stigma receptivity (full line) is given as the percentage of pollinated ﬂowers that set fruit; pollen viability (dotted line); sugar amount in nectar per flower (dashed line).

*Pollen viability:* No pollen viability was detected at bud stages (fl1 and fl2). During the first day of anthesis, the viability of the pollen grains reached 88–95 %. This proportion decreased at the end of the first day (1700 h., 66–72 %) and was lower the second day (45–48 %, Fig. 4). The percentage of pollen viability varied among sites (*F*_3,108 _=_ _2.80, *P* = 0.0435), whereas its decrease with time was similar (*F*_6,108 _=_ _0.61, *P* = 0.1257). The highest pollen viability was observed in Godomey and the lowest in Namougo (95 ± 3 % vs. 88 ± 7 % on the first day of anthesis).

*Stigma receptivity:* Stigma receptivity did not differ among sites (*F*_3 48 _=_ _0.33, *P* = 0.8013) nor among floral stages (*F*_2,48 _=_ _0.54, *P* = 0.5853). The stigmas were receptive just before (fl2) and during flower anthesis (Fig. 4). Pollen germination reached 68–74 % for well-developed buds and 74–80 % at flower anthesis.

**Figure 5.**
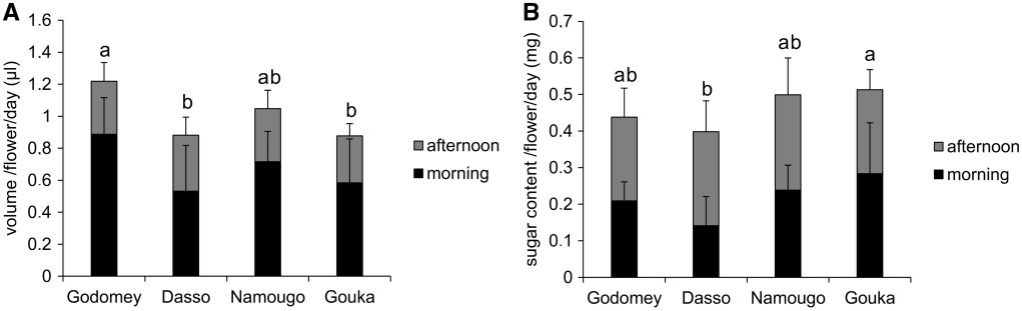
Nectar production of *Vitex doniana* ﬂowers on the four studied sites, according to time of day. (A) Volume of nectar produced per flower per day; (B) total sugar content in nectar per flower per day. Different letters indicate significant difference at *P *< 0.05 according to Tukey’s HSD post-hoc analysis.

*Nectar production*: The volume of nectar produced per flower per day varied among sites and was higher in Godomey (1.2 µL) compared with Dasso and Gouka (< 1 µl; *F*_3,192 _=_ _8.80, *P* < 0.0001, Fig. 5A). Similarly, mean sugar concentration in nectar differed among sites (*F*_3,192 _=_ _102.93, *P* < 0.0001). It was approximately 39 ± 17 % in Godomey, 41 ± 17 % in Dasso, 51 ± 11 % in Namougo and 45 ± 16 % in Gouka. Thus, the total sugar content in nectar was higher in Gouka (0.5 mg/flower) and lower in Dasso (< 0.4 mg/flower; *F*_3,192 _=_ _4.78, *P* = 0.0031, Fig. 5B). Regardless of site, the volume of nectar produced was higher in the morning than in the afternoon (*F*_1,192 _=_ _179.46, *P* < 0.0001, Fig. 5A), but the morning nectar had lower concentrations of sugars (29 ± 8 % vs. 58 ± 4 %; *F*_1,192 _=_ _2875.74, *P* < 0.0001). Nectar contained sucrose (39–58 %), glucose (23–35 %) and fructose (19–25 %).

### Visitor diversity, efficiency and fidelity

**Figure 6.**
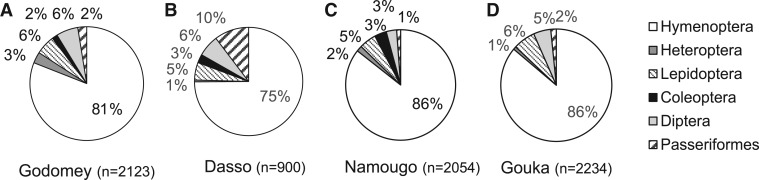
Visitor proportions on *Vitex doniana* recorded per site, on the four studied sites in (A) Godomey, (B) Dasso, (C) Namougo and (D) Gouka (*n* = total number of different visitors in 17 h of observation per site).

*Abundance and diversity of visitors*: During a total of 68 h of observation, we recorded 7311 insect visitors, belonging to 27 species (Table 3). We also recorded 207 bird visitors belonging to the Nectariniideae family (one species, the Splendid sunbird, *Cinnyris coccinigastrus*, Table 3). The numbers of visitors (over 17 h of observation at each site) was significantly lower in Dasso (total = 900) compared with the other sites (Fig. 6 and Table 3). The diversity of visitor fauna differed among sites (*X*^2 ^=^ ^374.801, d*f* = 15, *P* = 0) and included several orders and families: Hymenoptera (78–89 %), Lepidoptera (2–6 %), Heteroptera (1–3 %), Coleoptera (0–4 %), Diptera (2–8 %) and Passeriformes (1–7 %, Fig. 6). Hymenoptera were mainly represented by the families Apidae and Megachilidae (Table 3). Within Apidae, the native honeybees (*Apis mellifera adansonii*) were the main visitors, representing 26–40 % of the observed visitors over all sites, with a higher abundance in Godomey. *Xylocopa olivacea* individuals were also abundant (24–32 %), especially in Gouka. Individuals from *Megachile cincta* (44–68 %) and *Megachile rufipes* (19–35 %) were the main visitors from the Megachilidae family. Within the Syrphidae family, *Eumerus vestitus* (40–64 %) and *Allobaccha* sp. (29–36 %) were particularly abundant. The Dasso site was characterized by the highest abundance of the sunbird, *Cinnyris coccinigastrus* (*X*^2 ^=^ ^52.652, df = 3, *P* = 0, Table 3).

**Table 3.** Insect and bird visitors on *Vitex doniana* in the four studied sites. Most abundant visitors for the different sites are indicated in grey.

	Family	Species	Total number recorded	Godomey	Dasso	Namougo	Gouka
Hymenoptera	Apidae	*Xylocopa olivacea* Fabricius	955	263	119	282	291
		*Xylocopa nigrita* Fabricius	127	22	11	43	51
		*Apis mellifera adansonii* Latreille	1189	407	110	383	289
		*Ceratina (Pithitis) viridis* Guérin Méneville	544	173	30	160	181
		*Ceratina (Simioceratina)* sp. Latreille	151	29	12	48	62
		*Dactylurina staudingeri* Gribodo	359	111	34	101	113
		*Braunsapis ghanae* Michener	368	85	71	92	120
		*Amegilla albocaudata* Dours	148	34	18	41	55
	Megachilidae	*Megachile cincta* Fabricius	916	223	87	236	370
		*Megachile rufipes* Fabricius	529	132	94	166	137
		*Megachile torrida* Smith	452	131	64	116	141
		*Euaspis abdominalis* Fabricius	153	37	16	47	53
		*Chalicodoma maxillosa* Guérin Méneville	98	27	8	16	47
		*Anthidium* sp*.* Fabricus	85	45	0	27	13
	Halictidae	*Lipotriches* sp Gerstaecker	125	67	5	34	19
Heteroptera	Lygaeidae	*Graptostethus servus* Fabricius	131	33	3	41	54
Lepidoptera	Arctiidae	*Euchromia lethe* Fabricius	53	9	13	13	18
	Lycaenidae	*Axiocerses harpax* Fabricius	68	30	4	15	19
	Hesperiidae	*Borbo perobscura* Druce	104	34	24	15	31
	Sphingidae	*Cephonodes hylas virescens* Wallengren	55	12	3	19	21
Coleoptera	Tenebrionidae	*Alogista serricorne* Kolbe	93	28	15	50	0
	Chrysomelidae	*Podagrixena decolorata* Duvivier	35	5	8	22	0
Diptera	Tephritidae	*Dacus ciliatus* Loew	136	74	21	16	25
	Syrphidae	*Allobaccha* sp Curran	51	12	8	11	20
		*Eumerus* sp Fabricus	34	13	5	0	16
		*Eumerus vestitus* Bezzi	76	33	8	14	21
	Bombyliidae	*Eurycarenus* sp Loew	69	0	16	22	31
Passeriformes	Nectariniidae	*Cinnyris coccinigastrus* Latham	207	54	93	24	36

**Figure 7.**
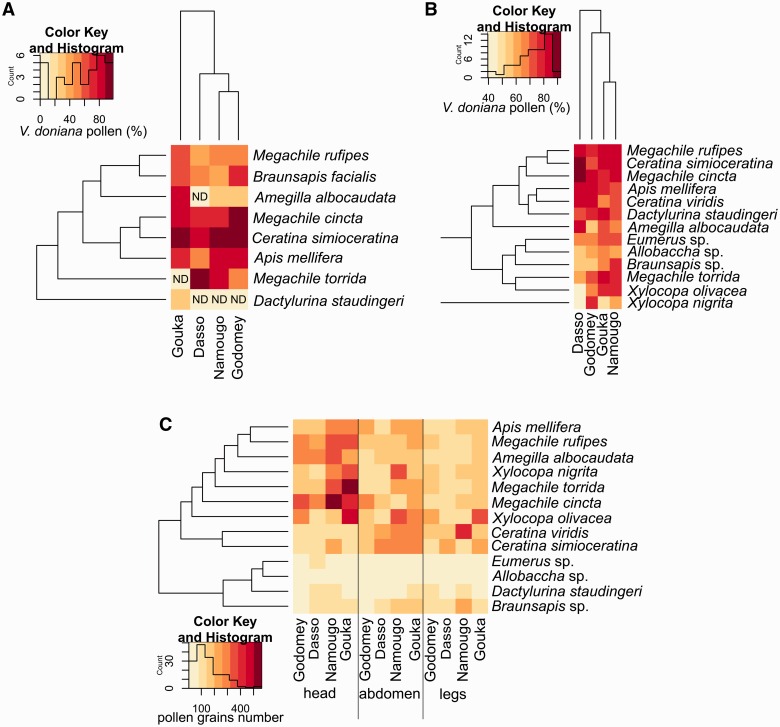
Insect visitor efficiency and fidelity on *Vitex doniana* recorded on the four studied sites. (A) Heatmap of the percentage of *V. doniana* pollen observed in the insect pollen loads according to insect species; (B) heatmap of the percentage of *V. doniana* pollen observed on the insect body according to insect species; (C) heatmap of the number of pollen grains on the different insect body parts according to insect species. ND, no data.

*Fidelity and pollen loads:* The proportion of *V. doniana* pollen in corbicular loads differed significantly among visitor species (*F*_3,33 _=10.40, *P* < 0.0001) and among sites (*F*_3,33_= 3.04, *P* = 0.0426), with a marginally significant interaction among sites and species (*F*_3,33 _=_ _2.20, *P* = 0.0270). The pollen loads from *Dactylurina staudingeri* contained little pollen from *V. doniana* (25 %, Fig. 7A). By contrast, 17 % of *Apis mellifera*, 40 % of *Megachile torrida* and 60 % of *Ceratina (Simioceratina)* individuals carried monospecific pollen loads of *V. doniana* (Fig. 7A).

*Pollen carrying capacity:* Insect visitors carried 42–87 % *V. doniana* pollen grains, depending on the insect species (*F*_14,206 _=_ _5.11; *P* < 0.0001) but not on the site (*F*_3,206 _=_ _1.62, *P* = 0.1849). *Vitex doniana* pollen grains constituted the majority of the carried pollen grains on 63 % of insects caught (Fig. 7B). The pollen grain numbers found on the insects differed significantly among body parts (*F*_2,618_= 28.48, *P* < 0.0001), among insect species (*F*_14,618 _=_ _87.91, *P* < 0.0001), and among sites, with the insects caught in the Guineo-Sudanian sites carrying more pollen than those caught in the Guineo-Congolian sites (*F*_3,618 _=_ _20.49, *P* < 0.0001, Fig. 7C).

The large bees like *Xylocopa* spp. and the medium-sized bees *Megachile* spp., *Apis mellifera* and *Amegilla albocaudata* used the lower petal as a ‘landing platform’ and during foraging, their heads contacted the anthers and stigma. They, therefore, received more pollen grains on their heads (39–65 % of total pollen load) than on their abdomens and legs (Fig. 7C). Small bees like *Braunsapis ghanae* or *Ceratina* spp., which directly plunged into the corolla tube, received more pollen grains (36–60 % of total pollen load) on their abdomens.

### Pollination trials

**Figure 8.**
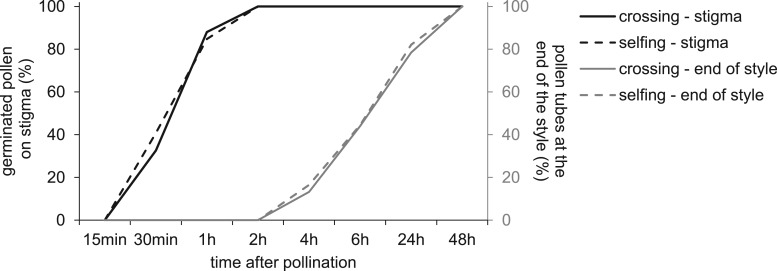
Cumulative percentage of germinated pollen on stigma (in black) and of pollen tubes reaching the end of the style (in grey) after hand cross-pollination (solid lines) or hand self-pollination (dashed lines) in *Vitex doniana*, according to time after pollination.

*Pollen germination and pollen tube growth*: Pollen germination was observed 30 and 60 min after pollination and pollen tubes reached the extremity of the style after 4–8 h (Fig. 8). Signs of fertilization in the ovary were observed 1–2 days after pollination. No differences were observed between self- and cross-pollinated styles for the number of pollen tubes reaching the ovary (*X*^2 ^=^ ^0.349, d*f* = 2, *P* = 0.8397).

**Figure 9.**
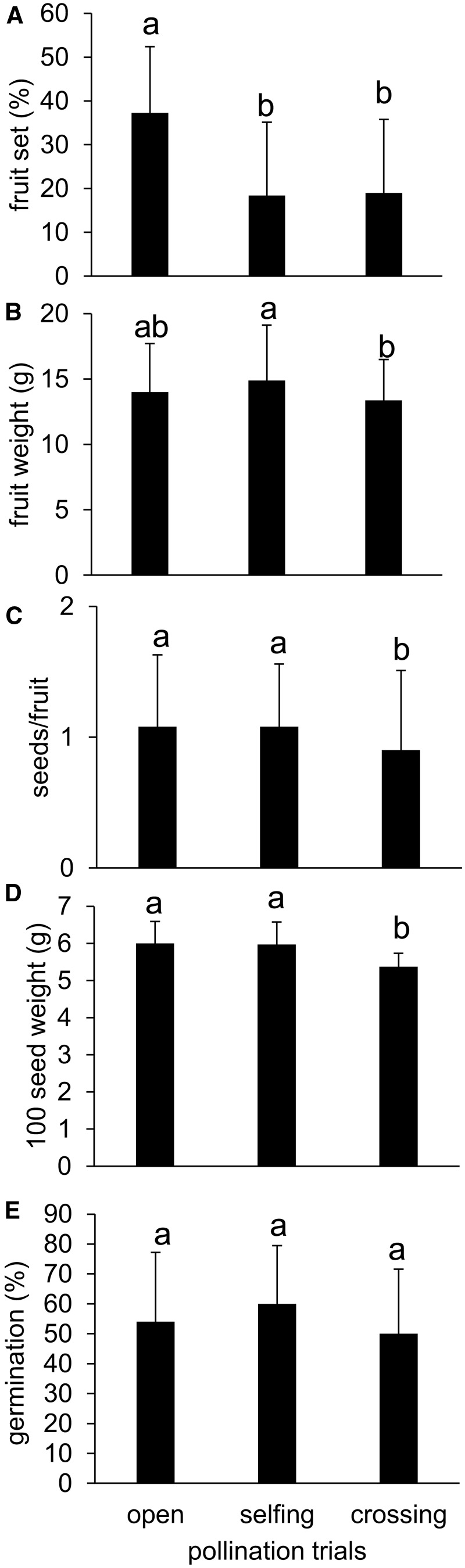
Fruit and seed parameters derived from pollination trials. (A) Fruit set, (B) fruit weight, (C) seed number per fruit, (D) weight of 100 seeds and (E) seed germination rate after open pollination (open), hand self-pollination (selfing) and hand cross-pollination (crossing) in *Vitex doniana*. Data are shown as means ± SD. Pollination trials followed by different letters are significantly different at *P *< 0.05 according to Tukey’s HSD post-hoc analysis.

*Fruit and seed production:* Since no differences were detected in fruit set (*F*_3,308 _=_ _2.33, *P* = 0.0744), seed set (*F*_3,308 _=_ _0.08, *P* = 0.9728) or fruit weight (*F*_3,308 _=_ _1.85, *P* = 0.1388) among sites, we pooled the results. No fruits were produced after spontaneous self-pollination (*SFI* = 0) at any site, indicating that pollen vectors are essential for pollination. Open-pollination treatment produced significantly more fruits (37 %, *F*_2,308 _=_ _39.54, *P* < 0.0001, Fig. 9A) than hand-pollination, but fruit weight was lower after cross-pollination compared with open- and self-pollination (*F*_2, 308 _=_ _4.63, *P* = 0.0104, Fig. 9B).

Seed set was significantly higher after open- and self-pollination than after cross-pollination (*F*_2, 308 _=_ _3.45, *P* = 0.0329, Fig. 9C). The weight of 100 seeds was lower after crossing compared with self- and open- pollination (*F*_2,36 _=_ _4.65, *P* = 0.0158, Fig. 9D). Self-compatibility indices were high (*SCI_f_* = 0.80 ± 1.26, *SCI_s_* = 1.22 ± 0.84). Germination of the resulting seeds did not differ between treatments (*F*_2, 36 _=_ _0.82, *P* = 0.4482) and reached approximately 50–62 % (Fig. 9E). No inbreeding depression was detected during the first developmental stages for seed set (−0.2 ± 0.16) or germination rate (−0.04 ± 0.1); indeed, selfed progeny performed better than outcrossed ones. Cumulative inbreeding depression reached *δ* = −0.04.

**Figure 10.**
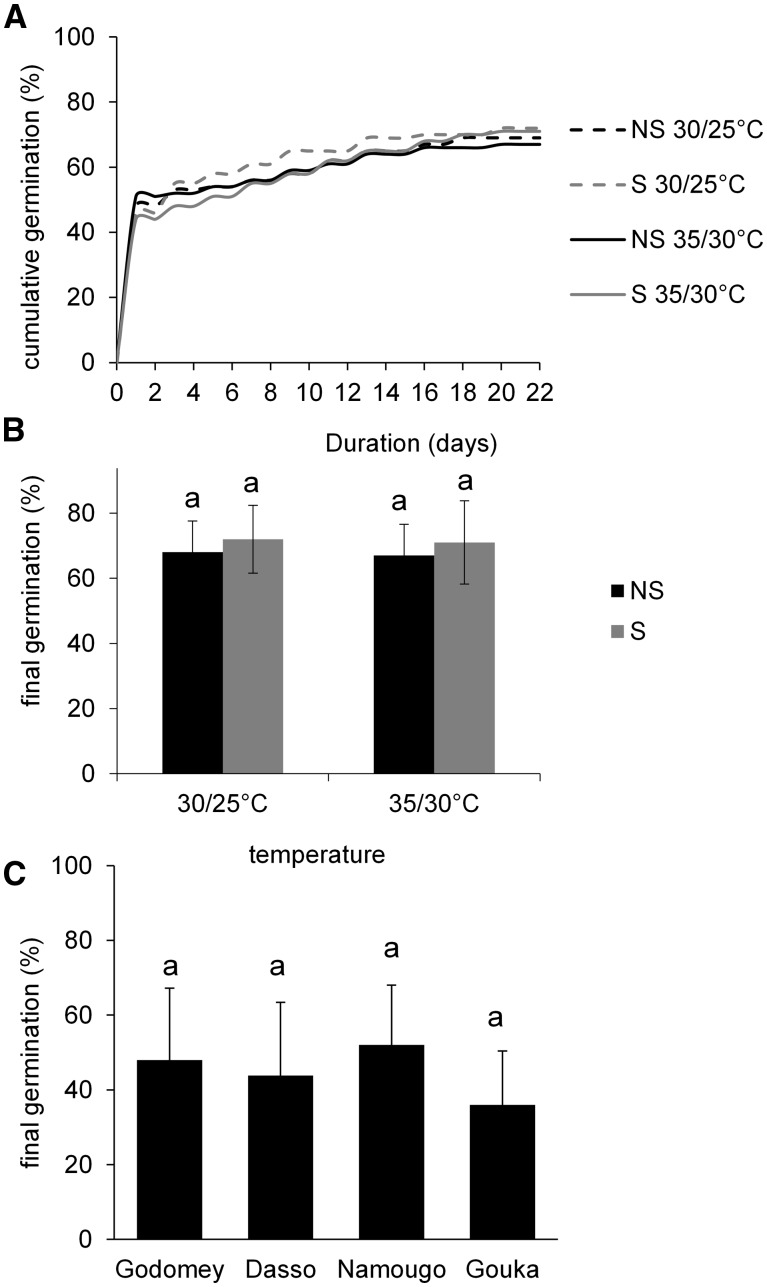
*Vitex doniana* seed germination. (A) Cumulative germination rate as a function of time; (B) final germination rate of seeds subjected to different temperatures (30/25 °C, 35/30 °C) and soaking treatments (NS: not soaking seeds, S: soaking seeds); (C) final germination rate of seeds sown in the four studied sites in Benin. Data are shown as means ± SD. Sites or treatments followed by different letters are significantly different at *P *< 0.05 according to Tukey’s HSD post-hoc analysis.

*Seed viability and germination:* Under controlled conditions, no germination occurred with pre-treated fruits but seeds that were extracted from the seed coat germinated within 2 days, regardless of pre-treatment (soaking or not) or temperature conditions (30/25 °C, 35/30 °C; Fig. 10A). After 21 days, germination rate reached 70 % and did not differ by pre-treatment (*F*_1,16 _=_ _0.03, *P *= 0.8715, Fig. 10B) or temperature conditions (*F*_1,16 _=_ _0.00, *P* = 0.10, Fig. 10B). Germination tests *in situ* resulted in a 36–52 % germination rate and did not differ among sites (*F_1,_*_377 _=_ _2.05, *P* = 0.1142, Fig. 10C).

## Discussion

### What is the floral phenology and morphology of *Vitex doniana*?

*Vitex doniana* flowering occurred once a year in the dry season (December–April) as generally found in other woody species from Sudanian and Sahelian zones (for example *Parkia biglobosa*, *Ziziphus mauritiana* and *Vitex fischeri*) while fruit maturation takes place during the rainy season which might favor seed dispersion, seed germination and sapling development (Ahenda 1999). Flowering patterns showed a shift according to sites and latitude (in December in the Guineo-Congolian sites, two weeks later in Guineo-Sudanian sites). These patterns resulted from the wetter conditions in Guineo-Congolian zone than in the Guineo-Sudanian one. This is congruent with the general assumption that the main determinants of phenology of tropical trees are temperature, insolation, rainfall and relative humidity (Ewédjè *et al.* 2015).

Flowering asynchrony was high at all levels, among sites, among trees and within trees. This low flowering overlap increases the attraction of pollinators, as floral rewards are offered during an extended period, and facilitates pollen exchange among flowers within a tree and among trees within a site. Thus with the observed efficient pollination rate, a low level of genetic differentiation could be expected within populations of this tree species (Ewédjè*et al.* 2015).

Several morphological traits of *V. doniana* flowers predispose them to selfing, including hermaphroditism, and a short anther–stigma distance (0.65–0.69 mm). Moreover, pollen viability is high on the day of anthesis and coincides with the stigma receptivity; this homogamy trait was also found on *Vitex negundo*, *V. fischeri* and *V. kenyensis* (Reddy and Reddi 1994; Ahenda 1999). Nevertheless, the divergent positions of the lobes of the stigma and the anthers could preclude spontaneous selfing. Such a floral morphology, combined with flowering asynchrony could suggest a mixed mating system, combining both geitonogamy and outcrossing.

### Is *Vitex doniana* efficiently pollinated by visitors?

The flower morphology and the production of floral rewards indicate that *V. doniana* could be attractive to visitors. The zygomorphic and tubular flowers present the typical gullet blossom of the genus *Vitex*, with a large inferior petal where insects can land. The morphology also suggests that most pollen-bearing insects likely contact the stigmas during a visit and could thus efficiently contribute to pollination.

Most flowers of *V. doniana* opened as soon as an insect or bird visitor touched any petal. This stage of flower (fl3) coincides with the peak of nectar production (0.75–0.89 µL), which might be an important reward. As with many Lamiaceae species, nectar is offered at the base of the corolla tube (Faegri and van der Pijl 1979; Jorge *et al.* 2015). The sugar concentration of nectar (39–51 % sugars), negatively correlated with the volume, remained high in all sites and during the entire flower life span. Such a high sugar concentration mainly attracts nectar-feeding insects. Birds usually prefer more dilute but more abundant nectar (Kulloti *et al.* 2011). In addition to nectar, *V. doniana* produced copious pollen (15 000–16 000 pollen grains per flower), which is attractive for pollen-collecting insects. *Vitex doniana* flowers, therefore, appear extremely attractive to insect visitors. Indeed, the species is considered a valuable melliferous plant and honey resource, and trees are sometimes chosen for hanging bark beehives (Djonwangwe *et al.* 2011; Yédomonhan *et al.* 2012; Dukku 2013).

We found that *V. doniana* flowers were extensively visited by insects and birds from early in the morning to late afternoon. *Vitex doniana* was visited by 27 insect species belonging to 13 families from five orders, and by one species of sunbird from the family Nectariniidae. It thus has a generalist pollination system. Other Lamiaceae species (*Salvia* spp., *Leonurus* spp., etc.) are also pollinated by both birds and insects (Proctor *et al.* 1996; Wester and Claβen-Bockhoff 2006; Kulloti *et al.* 2011). However, birds remained marginal visitors for *Vitex doniana* since they accounted only for 3 % of the observed visitors.

The Hymenoptera were the most abundant visitors at the four sites. Apideae species (45–56 % of visitors), mainly represented by *Apis mellifera* and *Xylocopa* spp., were the main visitors. Similar insect guilds were observed on *Vitex negundo* (Reddy and Reddi 1994)*, V. fischeri* and *V. kenyensis* (Ahenda 1999). Pollen load analyses showed that most bee individuals recorded were foraging on *V. doniana* even if other plant pollen grains were found in their corbicular loads. Fidelity to *V. doniana* pollen differed significantly among species and among sites. Individuals of the species *Apis mellifera*, *Ceratina* sp., *Megachile cincta* and *Megachile torrida* collected *V. doniana* pollen in higher proportion than did other species. Pollen grains carried by the most abundant *V. doniana* visitors were predominantly those of this plant species whatever the site. The high proportions of *V. doniana* pollen grains on insect bodies indicated that these four species might be more efficient pollinators. Hymenoptera are prevalent pollen-bearing insect visitors, which typically carry large quantities of pollen on their bodies (and in their pollen loads).

### What is the breeding system of *Vitex doniana*?

Most fruits contained only one seed for four ovules. As with many Lamiaceae species, including others from the genus *Vitex*, three out of the four ovules usually abort in response to post-zygotic competition within the developing fruit (Ahenda 1999; Eyog-Matig *et al.* 2002; Ky 2008).

In controlled hand-pollinations, *V. doniana* produced similar or even higher numbers of fruit and seed after self- than after cross-pollinations, indicating that the species is self-compatible, as observed in other species of the genus such as *Vitex negundo* (Reddy and Reddi 1994). Being a self-compatible plant with many flowers simultaneously open, the species might experience selfing both by autogamy and by geitonogamy, mediated by pollinators. However, seed set and seed viability did not differ among self- and cross-pollinations, indicating that the species does not suffer from inbreeding depression.

Despite the observed self-compatibility, *V. doniana* did not produce any fruits and seeds after spontaneous selfing (*SFI *= 0). The species, therefore, requires pollen vectors for its pollination.

### What are the best germination conditions for *Vitex doniana*?

This species has a hard seed coat, which causes very low germination rates (Ahoton *et al.* 2011). Indeed no germination was observed without extracting seeds from the fruits. Moreover, germination generally occurs over a long period (Ahoton *et al.* 2011; N’Danikou *et al.* 2014). The hard seed coat prevents the entry of water and oxygen, which could break seed dormancy (Mapongmetsem 2006; Ahoton *et al.* 2011). Germination is most successful with fresh seeds. Many seed treatments have been suggested to break dormancy and improve germination, without any conclusive recommendations. In the literature, a higher germination rate (50–58 %) was obtained with the dormancy-breaking treatment of alternation of 8 h sun-drying and 1 h soaking in tap water for 3 days (N’Danikou *et al.* 2014), or with physical shock (Ahoton *et al.* 2011). Our results in the field (50 % germination) were similar and also probably due to the alternation to drying and soaking, with suitable water uptake by water-impermeable layers due to palisade dislocation. Furthermore, alternately drying and soaking seeds would have induced a temperature fluctuation within the seed coat that is reported to promote germination. The germination rates obtained under controlled conditions in our study were significantly higher (70 %) and faster (< 21 days) than all previous studies. Time to the first germination (2 days) was shorter than that obtained by Ahoton *et al.* (2011, 11 days) or N’Danikou *et al.* (2014, 18 days). Germination rates were also higher than those obtained for scarified seeds of other related *Vitex* species, as a previous study found 25–50 % germination for *V. keniensis* and *V. fischeri* (Ahenda 1999) or 6 % for *V. madiensis* (Mapongmetsem 2006). Our high germination rates most probably resulted from the prior destruction of the almost impermeable and hard seed coat, which delays and inhibits seed germination. Soaked and not-soaked seeds displayed no significant differences under the incubation conditions of 30/25 °C and 35/30 °C, probably due to the relatively high water content of seeds freshly extracted from fruits. These incubation temperatures corresponded to the soil temperatures that the dispersed seeds might experience in their natural environment (N’Danikou *et al.* 2014; Ewédjè *et al.* 2015).

### Implications for conservation strategies

While not measured directly during this study, the reproductive traits of the species are most likely to be impacted by extensive leaf harvesting, which delays or prevents flowering of trees, which reduce fruit and seed availability. Even if the flowering pattern of this species is influenced by climatic variables, as found for many other plant species, the regularly delayed or cancelled flowering of trees from the site Dasso suggests that leaf collection puts significant biological stress on the reproductive performance of *V. doniana* (Oumorou *et al.* 2010). Leaf harvesting induces low flower production and extends the flowering shift among trees. To make matters worse, when collecting new leaves, harvesters cut the entire twigs with floral buds. Moreover, to facilitate leaf collection, branches and even trees are cut down (N’Danikou *et al.* 2015).

Nevertheless, fruit and seed sets in exploited sites, such as Dasso, remained similar to those in protected sites (Namougo and Gouka), showing that pollination remained sufficient to ensure suitable reproductive success. The major threat seems thus not a decrease of reproductive success at the flower level but the decrease of flower production. A decrease in flower production induced a lower number of fruits. Moreover, as fruits are harvested, there are no seeds left for natural recruitment. Further studies on *V. doniana* population biology would help to evaluate the real impact of overexploitation on local population survival. Moreover, studies are ongoing to investigate conservation strategies in Benin.

The low level of natural seed germination and poor recruitment (Ahoton *et al.* 2011, N’Danikou *et al.* 2015) can be compensated by the high germination rates obtainable by destruction of the hard seed coat. Apart from vegetative propagation (Oumorou *et al.* 2010; Mapongmetsem *et al.* 2012; Sanoussi *et al.* 2012; Achigan-Dako *et al.* 2014), the enrichment of residual populations of *V. doniana* could be fostered using imbibed seeds extracted from fruits. Survival rates in experimental plantations are generally good, reaching 80–90 % after 3 years (N’Danikou *et al.* 2015). As multiplication by seeds is a tractable conservation method, a strategic action might be to organize both *ex situ* and *in situ* conservation approaches, studying the possibilities for cultivation and domestication in orchards or in managed agroforestry systems (Oumorou *et al.* 2010; Vodouhè *et al.* 2011; N’Danikou *et al.* 2015). Producing *V. doniana* in horticultural or in peri-urban settings would enlarge the resource base and keep pressure off natural stands. Based on the current demand for *V. doniana* products and the intensity of the harvest, cultivation would be the best option to sustain the species (N’Danikou *et al.* 2015). Authorities could encourage local people to plant, protect and promote natural regeneration in their fields or in their home gardens (Oumorou *et al.* 2010). The protection, cultivation or domestication of this species and its integration into diverse agroforestry systems are important components of a strategy for the improvement of land use in Africa (Vodouhè *et al.* 2011).

## Conclusion

*Vitex doniana* is a hermaphroditic, homogamous and self-compatible species, presenting a predominant selfing through geitonogamy. Moreover, *V. doniana* trees are abundantly visited by a wide array of insect and bird species. These reproductive traits assure sexual reproduction even for isolated and distant trees in small, remnant populations. These pollination and breeding characteristics offer the required conditions to develop successful conservation strategies. This study proved that reproductive biology is primordial before any conservation strategy planning for poorly studied species.

## Sources of Funding

This work was founded by the Université catholique de Louvain (ADRI) with a Ph.D. grant to V. Sinébou.

## Contributions by the Authors

V.S. performed all the experiments both in the field (Benin) and in the lab (Belgium) as a part of her PhD dissertation. M.Q. performed the statistical analyses, drawn the figures and co-wrote the manuscript. B.C.A. supervised the field experiments in Benin. A-L J. designed the experiments, analysed the results and wrote the manuscript. All authors approved the final version of the manuscript.

## Conflict of Interest Statement

None declared.
